# OPTIMIZING OLDER ADULT BLOOD PRESSURE SCREENING IN A COMMUNITY SETTING BY INTERPROFESSIONAL STUDENTS

**DOI:** 10.1093/geroni/igac059.1978

**Published:** 2022-12-20

**Authors:** Erin Miller, Rima Charara, Ali Turfah, Jennifer Mendez

**Affiliations:** Wayne State University School of Medicine, Detroit, Michigan, United States; Wayne State University Eugene Applebaum College of Pharmacy and Health Sciences, Dearborn, Michigan, United States; University of Michigan School of Public Health, Ann Arbor, Michigan, United States; Wayne State University, Toronto, Ontario, Canada

## Abstract

A novel program was implemented in an effort to optimize the blood pressure taking skills of medical and pharmacy students, in collaboration with older individuals in Detroit. Under the supervision of Interprofessional faculty, pharmacy and medical students provided blood pressure screenings at the popular Riverwalkers program, a community exercise program designed for older Detroiters. Prior to volunteering at the program, students were required to complete the AMA blood pressure modules to develop proficiency in blood measurement best practices. Over the course of two months, 72 students provided blood pressure screenings for 79 older individuals during 266 distinct encounters. We believe this program can serve as a model for other professional schools, as it efficiently accomplishes several distinct

